# Chemical Composition of the Essential Oils of Three Popular *Sideritis* Species Cultivated in Greece Using GC-MS Analysis

**DOI:** 10.3390/biom13071157

**Published:** 2023-07-20

**Authors:** Eleftheria H. Kaparakou, Dimitra Daferera, Charalabos D. Kanakis, Efstathia Skotti, Maroula G. Kokotou, Petros A. Tarantilis

**Affiliations:** 1 Laboratory of Chemistry, Department of Food Science and Human Nutrition, Agricultural University of Athens, Iera Odos 75, 11855 Athens, Greece; elek@aua.gr (E.H.K.); daferera@aua.gr (D.D.); chkanakis@aua.gr (C.D.K.); mkokotou@aua.gr (M.G.K.); 2 Department of Food Science and Technology, Ionian University, Terma Leoforou Vergoti, 28100 Argostoli, Greece; efskotti@ionio.gr

**Keywords:** *Sideritis*, mountain tea, GC-MS analysis, essential oils

## Abstract

(1) Background: The essential oils (EOs) of *Sideritis* L. have attracted great interest due to their pharmacological activities and potential applications in the cosmetic and perfume industries. The aim of this work was to study the EO chemical composition of three of the most popular, in Greece, mountain tea species: namely, these include *Sideritis scardica*, *Sideritis raeseri*, and *Sideritis syriaca*. (2) Methods: The EOs were obtained from the aerial parts of three *Sideritis* species that were cultivated in various regions of Greece by hydrodistillation, and the chemical composition was studied by gas chromatography–mass spectrometry (GC-MS) analysis. (3) Results: The EOs of the *Sideritis* species—*S. scardica* (SSC1, SSC2, SSC3), *S. raeseri* (SR1, SR2, SR3), and *S. syriaca* (SS1, SS2, SS3)—were analyzed by GC-MS, and they showed both qualitatively and quantitatively high variation in their chemical composition. (4) Conclusions: The EOs of *S. scardica* and *S. raeseri* from three different regions of Greece, and the *S. syriaca* from three different localities of Crete Island in Southern Greece, showed high chemical variability. Although 165 different components were found to be present in the nine samples through GC-MS analysis, only 7 (1-octen-3-ol, linalool, *trans*-pinocarveol, *p*-mentha-1,5-dien-8-ol, *α*-terpineol, myrtenol, and verbenone) were common components in the nine EOs, which were identified to be highly variable in different percentages among the samples. Even the EOs of SS1 and SS2, which were cultivated nearby, showed different GC profiles. The composition variation observed might be attributed to differentiations in the soil and climatic conditions.

## 1. Introduction

Mountain tea, which belongs to the genus *Sideritis* spp. (Lamiaceae family), and is also known as Dioscorides siderite, is very popular in the Mediterranean area for its use as an herbal tea. The genus name, which comprises more than 150 species and several subspecies, derives from the Greek word for “iron”, thanks to the plant’s healing action against wounds caused by iron weapons [1]. Siderites, Olympus tea, malotira, good sleeper, Malevos tea, and Taygetus tea, are some of the local names used in Greece to describe mountain tea. In the Mediterranean region and the Balkan Peninsula, many locally endemic species of the genus *Sideritis* exist. In Greece, apart from the species *S. scardica* and *S. raeseri,* which are the most widespread, several species of mountain tea can be found, most of which are collected wild and consumed on a local scale, and they are restricted to narrow mountainous areas or (uniquely to) islands, such as *S. syriaca* in Crete Island and *S. euboea* in Evia Island [2].

The therapeutic use of *Sideritis* species was first mentioned by Dioscorides in his book “De Materia Medica” [3]. Over the years, *Sideritis* species have found applications in Mediterranean traditional medicine [4] for their anti-inflammatory, antirheumatic, and antimicrobial activities [1,5]. Several investigations into plants belonging to the genus *Sideritis* L. have revealed a plant-derived source of particular pharmacological and nutritional interest [5].

Essential oils are isolated from aromatic plants using a variety of methods, including hydrodistillation, solvent extraction, cold pressing, and supercritical fluid extraction [6]. EOs find a wide area of applications in the food industry, as they are used as food flavors, natural additives, and in the preservation of foods because of their antioxidant and antimicrobial properties [7]. EOs extracted by several *Sideritis* species have been studied for their antimicrobial [8,9], antioxidant, anti-inflammatory, and antiproliferative action [9], while their antidiabetic, antiurease, cytoprotective, nematicidal, analgesic, allelopathic, and antirust effects have not been studied sufficiently [10]. A very recent review article summarizes and discusses the chemical composition and pharmacological activities of EOs of the genus *Sideritis* L. [10].

In that work presented, the pharmacological properties of *Sideritis* species that have been studied until now have mainly focused on the antibacterial, antifungal, and antioxidant activities. Specifically, the EO of *S. raeseri* (main compounds: geranyl-*p*-cymene (25.08%), geranyl-γ-terpinene (15.17%), geranyl-linalool (14.04%), *γ*-elemene (5.73%)) showed low antibacterial activity, compared to positive controls, gentamycin and ciproxin. Also, the EO of *S. scardica* (main compounds: hexadecanoic acid (12.92%) and myristicin (5.23%)) showed low activity in Gram-positive and Gram-negative bacteria when compared to the positive control (gentamycin). However, the EO of *S. syriaca* (main compounds: carvacrol (33.68%), *β*-(E)-caryophyllene (8.47%), *β*-phellandrene/limonene (6.84%), and bicyclogermacrene (5.29%)) has shown high antibacterial activity. This EO was shown to be rich in carvacrol (33.68%), which is a widely noted antibacterial agent. The EO of *S. raeseri* was also studied for its antifungal activity against *Saccharomyces cerevisiae* and *A. niger*, and the MIC values were significantly higher than those of the control (voriconazole). In addition, the EO of *S. syriaca*, which is characterized by the presence of a high carvacrol percentage (33.68%), presented the strongest effect against pathogenic microorganisms. Furthermore, the EO of *S. raeseri* showed low antioxidant activity when studied by DPPH and ABTS assays [10].

The aim of the present study was to investigate and compare the chemical composition of three of the most popular *Sideritis* species in Greece: *Sideritis scardica*, *Sideritis raeseri*, and *Sideritis syriaca*, which are mainly cultivated at small scales in Greece and Bulgaria, as previously reported [11], and have not been studied enough until now. According to the existing literature, there is only one reference where an EO of a *S. scardica* from Greece has been studied, but it originated from a different area of our study: four references where the EOs of *S. syriaca* have been studied, of which two of them concerned wild samples, and six references where the EOs of *S. raeseri* have been studied, which three of them concerned cultivated samples from different areas of our study [10]. With our research, we are aiming to reinforce the literature regarding the chemical composition of the EOs from the *Sideritis* species, which are cultivated in Greece in different geographical areas, such as *S. scardica* or Olympus tea, which is possibly the most well-known herbal/mountain tea in Greece and the Balkan Peninsula.

## 2. Materials and Methods

### 2.1. Chemical and Reagents

Diethyl ether and magnesium sulfate (anhydrous) were purchased from Sigma-Aldrich (Taufkirchen Deutschland and St. Louis, MO, USA, respectively).

### 2.2. Plant Material

**Figure 1:**
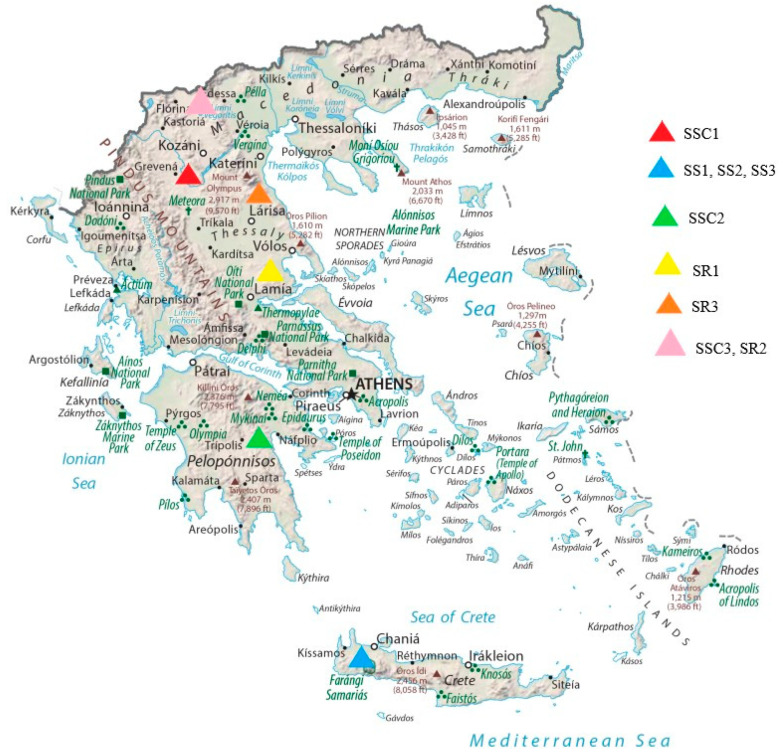
Map of the origin of samples of *Sideritis* species.

For this study, the aerial parts of three *Sideritis* species were harvested from different regions of Greece. The first sample of *S. scardica* (SSC1) was cultivated in the area of Mount Olympus in Central Greece (Olympus tea), while the second sample of *S. scardica* (SSC2) was cultivated in the area of Mount Mainalo in Peloponnesos; finally, the third sample of *S. scardica* (SSC3) was cultivated in the area of Kastoria in Northern Greece. The first sample of *S. raeseri* (SR1) was cultivated in the foothills of Mount Othrys in Central Greece, the second sample of *S. raeseri* (SR2) was cultivated in the area of Kastoria in Northern Greece, and the third sample of *S. raeseri* (SR3) was cultivated in the area of Elassona, Larissa, in Central Greece. The three samples of *S. syriaca* were cultivated in Crete island: the first sample of *S. syriaca* (SS1) was cultivated in the southern part of the White Mountains (Lefka Ori), the second sample of *S. syriaca* (SS2) was cultivated in the area of Anopoli Sfakion near to White Mountains (Lefka Ori), and the third sample of *S. syriaca* (SS3) was cultivated in the area of Omalos Chanion (Figure 1).

Voucher specimens were deposited (No 012276: SSC1, No 012279: SSC2, No 012293: SSC3, No 012277: SR1, No 012294: SR2, No 012295: SR3, No 012278: SS1, No 012291: SS2, and No 012292: SS3) and maintained at the Herbarium of the Agricultural University of Athens (Table 1).

### 2.3. Isolation of the Essential Oil

The fresh aerial parts of these plants were subjected to hydrodistillation for 3 h using a Clevenger-type apparatus. The yields of EOs (Table 2) for these 9 samples ranged from 0.01% (*v*/*w*) (SS3) to 0.10% (*v*/*w*) (SR3). Due to their extremely low oil yield, EOs were obtained by liquid–liquid extraction using diethyl ether. The diethyl ether phase was then concentrated under a gentle flow of nitrogen stream, and the resulting solutions of oils were dried over anhydrous magnesium sulfate and stored in the freezer.

### 2.4. Gas Chromatography–Mass Spectrometry (GC-MS)

The EOs were analyzed using a gas chromatography instrument (SCION) coupled with a mass spectrometer detector and an autosampler (CP-8400) (Bruker, Carteret (NJ), USA). The capillary column used was OPTIMA-5 MS, 30 m × 0.25 mm, 0.25 μm. Helium (He) was used as the carrier gas, with a flow rate of 1.0 mL/min. The temperature at the injector was 220 °C and at the ionization source was 230 °C. The source was operated with an electric voltage of 70 eV. The analysis program, which had a total duration of 73.33 min, involved a rise in the temperature of the column, which was initially at 60 °C and increased gradually up to 250 °C at a rate of 3 °C/min and at a rate of 5 °C/min up to 300 °C. The volume of the sample to be analyzed was 1 μL.

### 2.5. Identification of the Components of the Essential Oils

The identification of volatile components of EOs was performed by comparing the mass spectra of the components with those from NIST and Adams mass spectral libraries and by comparing literature and estimated arithmetic (retention) indices that were determined using mixture of homologous series of normal alkanes from C_7_–C_24_ in n-hexane under the same conditions.

## 3. Results

**Figure 2:**
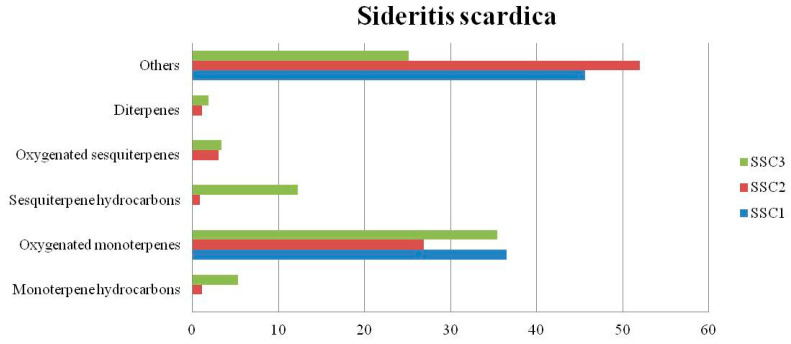
Group components (%) of *Sideritis scardica* EOs.

**Figure 3:**
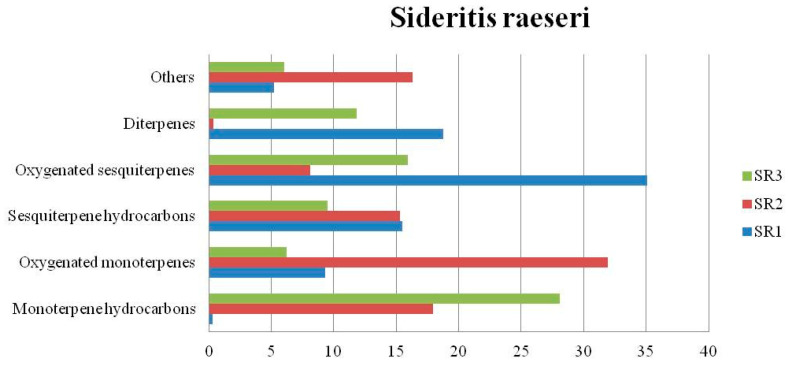
Group components (%) of *Sideritis raeseri* EOs.

**Figure 4:**
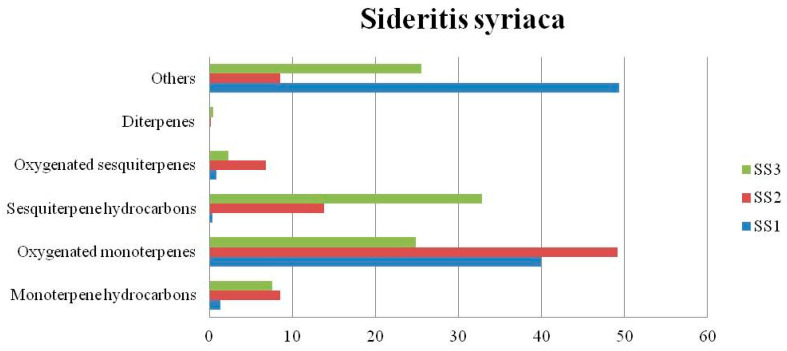
Group components (%) of *Sideritis syriaca* EOs.

The chemical composition (%) of the EOs of the nine *Sideritis* samples are summarized in Table 3. In the *Sideritis scardica* EOs (SSC1 and SSC2), others constituted the main group of components (Figure 2) yielded in a total amount of 45.65% and 52.02%, respectively, while for the essential oil of SSC3, the group of components known as oxygenated monoterpenes was found as the predominant amount in a percentage of 35.46%. In the *Sideritis raeseri* EOs (SR1, SR2, and SR3), considerable variations were observed (Figure 3). Oxygenated sesquiterpenes constituted the main group of components found in SR1 in a total amount of 35.1%, while in the SR2 and SR3, oxygenated monoterpenes and monoterpene hydrocarbons were estimated in a total amount of 31.91% and 28.12%, respectively. Considerable variations were also observed in the *Sideritis syriaca* EOs (Figure 4). In the essential oil of SS1, the main group of components was others (49.38%), because of the presence of 1-octen-3-ol (17.90%) and hexanol (16.23%) in a high percentage. In the SS2, oxygenated monoterpenes constituted the main group of components yielded in a total amount of 49.13%, while sesquiterpene hydrocarbons (32.87%) constituted the main group of components found in the SS3.

In the EOs of SSC1 (Table 3), 25 components were estimated, among which eugenol (21.52%) was the predominant component found, followed by benzene acetaldehyde (16.42%). In the EO of SSC2, 30 components were identified, and the major one was 1-octen-3-ol (37.28%). A total of 32 components were estimated in the EO of SSC3, with carvacrol (19.91%) and 1-octen-3-ol (13.99%) being the principal components.

A total amount of 106 components were observed in the EO of SR1, and *(Z)*-9-octadecen-1-ol (12.00%) was found to be the predominant component. The chemical composition of SR2 yielded 62 components, with *β*-pinene (7.50%), 1-octen-3-ol (6.14%) and α-pinene (5.33%) possessing higher percentages among the others. In the essential oil of SR3, 59 compounds were identified, and geranyl-*α*-terpinene (5.84%) and bicyclogermacrene (5.82%) were present at almost equal percentages.

The chemical composition of the essential oil of SS1 consisted of 48 compounds, where the principal compounds were 1-octen-3-ol (17.90%) and hexanol (16.23%). In the EO of SS2, 46 compounds were identified, and carvacrol (19.75%) was the predominant one. In addition, the essential oil of SS3 yielded 57 components, with *(E)*-caryophyllene (10.18%) and hexanol (9.84%) being yielded as the predominant components.

**Figure 5:**
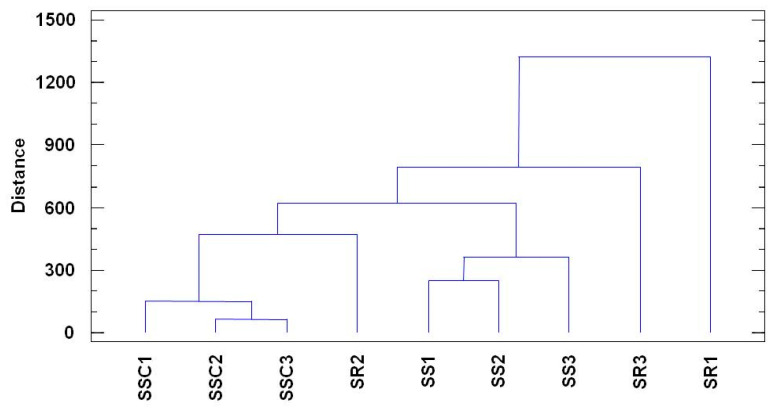
Dendrogram of the chemical variations and relationships between the *Sideritis* species (Statistical analysis was conducted through the package Statgraphics, which was performed using Word’s method).

Τhe chemical variation and relationship between the *Sideritis* species are presented in the dendrogram (Figure 5). As can be observed, based on their chemical composition the samples were initially divided into two groups. The 1st group included the sample SR1, and the 2nd group included all the other samples (SSC1, SSC2, SSC3, SR2, SR3, SS1, SS2, and SS3). Then, the samples of the 2nd group were divided into subgroups; the 1st subgroup had only one sample—SR3—and 2nd subgroup included the rest of the other samples (SSC1, SSC2, SSC3, SR2, SS1, SS2, and SS3).

In this second subgroup, the samples were separated into smaller subgroups: one subgroup (3rd subgroup) consisted of the samples SS1, SS2, and SS3, and another subgroup included the samples SR2, SSC1, SSC2, and SSC3 (4th subgroup). The 3rd subgroup samples were also divided into smaller subgroups: the 5th subgroup included SS1 and SS2, and the 6th subgroup included SS3; the 4th subgroup samples were divided into the 7th subgroup—SR2—while the 8th subgroup included SSC1, SSC2, and SSC3. Finally, the 8th subgroup samples were divided into a 9th subgroup—including SSC1—and a 10th subgroup—including SSC2, SSC3.

According to these results, the EO of the SR1 was differentiated due to its chemical composition from the rest of the eight EOs. Both of the other two EOs of the *S. raeseri* samples were quite different from the rest. The EOs of *S. syriaca* (SS1, SS2, and SS3) and *S. scardica* (SSC1, SSC2, and SSC3), showed more similar chemical composition, especially the EOs of SS1 and SS2, as well as SSC2 and SSC3.

**Figure 6:**
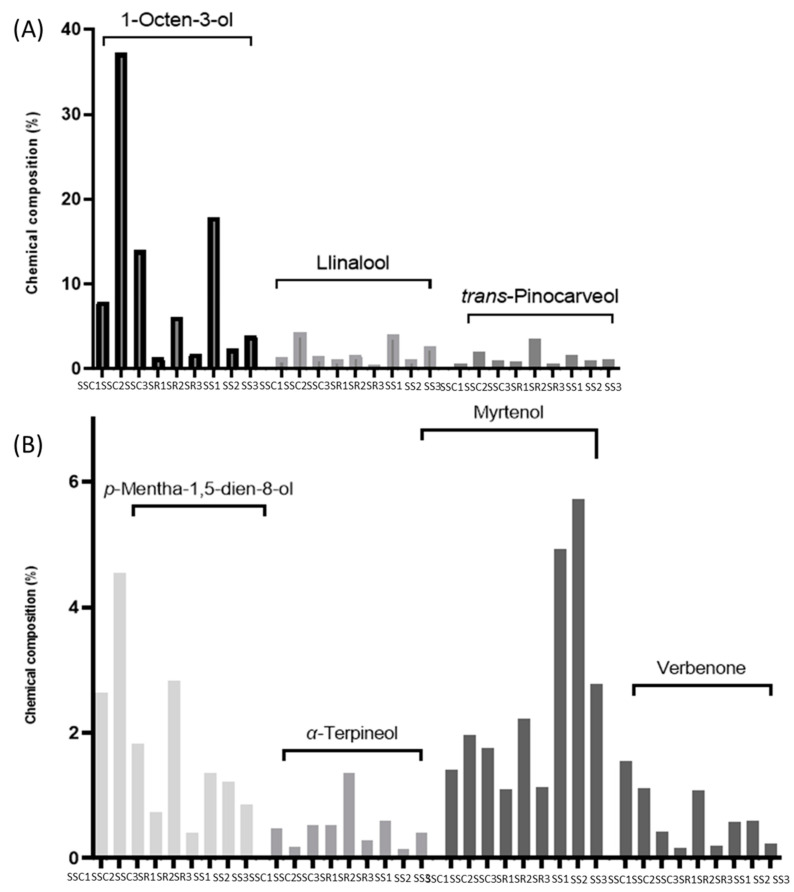
Variation of the seven components in the nine EOs. (subfigure (**A**) 1-octen-3-ol, linalool, *trans*-pinocarveol; subfigure (**B**) *p*-mentha-1,5-dien-8-ol, α-terpineol, myrtenol, and verbenone). SSC1: *S. scardica* from Olympus; SSC2: *S. scardica* from Mainalo; SSC3: *S. scardica* from Kastoria; SR1: *S. raeseri* from Othrys; SR2: S. raeseri from Kastoria; SR3: *S. raeseri* from Elassona; SS1: *S. syriaca* from Lefka Ori; SS2: *S. syriaca* from Anopoli Sfakion; SS3: *S. syriaca* from Omalos. Graphs were created using GraphPad Prism 9.2.0.

In the nine samples studied, 165 different compounds were found in total. However, only seven of them were identified to be present in all of the nine samples. Figure 6 depicts the contents of the seven common components, namely, 1-octen-3-ol, linalool, *trans*-pinocarveol, *p*-mentha-1,5-dien-8-ol, α-terpineol, myrtenol, and verbenone. In five out of the nine samples, 1-octen-3-ol was estimated as the most common component (>6%) (Figure 6A). All of the rest of the common components (linalool, *trans*-pinocarveol, *p*-mentha-1,5-dien-8-ol, α-terpineol, myrtenol, and verbenone) were found at percentages that were lower than 6% (Figure 6A,B).

## 4. Discussion

### 4.1. Sideritis scardica

The EOs of the *S. scardica* from three different regions of Greece (Central—SSC1, Southern—SSC2, and Northern—SSC3) showed variable chemical composition. The common components of these three EOs were benzaldehyde; 1-octen-3-ol; benzene acetaldehyde; linalool; *p*-mentha-1,5-dien-8-ol; *trans*-pinocarveol; *α*-terpineol; myrtenol; verbenone; and eugenol.

Kouklina et al. have studied EOs of *S. scardica* from two different locations in Greece and noticed the presence of *α*-pinene (8.20%, 17.80%), *β*-pinene (12.80, 13.10%), bicyclogermacrene (6.6%, 7.1%), and D-germacrene (6.60%, 2.20%) [13], which were also identified in our samples: *α*-pinene (1.12% in the SSC2 and 3.86% in the SSC3), *β*-pinene (1.43% in the SSC3), bicyclogermacrene (3.33% in the SSC3), D-germacrene (0.87% in the SSC2 and 3.80% in the SSC3). The presence of *α*-pinene (4.40–25.1%) and *β*-pinene (2.80–18.00%) were also observed in another study of the EOs of six populations of *S. scardica* that originated from Bulgaria [14]. The major component of the essential oil of SSC2 was 1-octen-3-ol (37.28%). This component was also identified in the EOs of the SSC1 and SSC3 in a percentage of 7.91% and 13.99%, respectively. These results are in accordance with a previous study, which reported the presence of 1-octen-3-ol in a range of 6.20–29.8% [15]. In addition, Trendafilova et al. reported the presence of 1-octen-3-ol (2.3–8%) in the EOs of *S. scardica* that originated from Bulgaria [14].

### 4.2. Sideritis raeseri

The EOs of the *S. raeseri* from three different regions of Greece (Central—SR1 and SR3 and Northern—SR2) showed different chemical composition. The common components detected in these three EOs were α-pinene; 1-octen-3-ol; linalool; nopinone; trans-pinocarveol; cis-verbenol; pinocarvone; terpinen-4-ol; α-terpineol; myrtenol; verbenone; trans-carveol; carvone; carvacrol; (E)-caryophyllene; (E)-β-farnesene; D-germacrene; bicyclogermacrene; spathulenol; caryohyllene oxide; α-bisabolol; benzyl benzoate; geranyl-p-cymene; hexadecanoic acid; and (Z)-9-Octadecen-1-ol.

Previous studies have reported the presence of *α*-pinene and *β*-pinene [8,14], which were also identified in our EOs: *α*-pinene (0.14% in the SR1, 5.33% in the SR2, and 4.46% in the SR3), *β*-pinene (7.50% in the SR2 and 3.00% in the SR3). In our samples, *(Z)*-9-octadecen-1-ol was also identified in a percentage of 12.00% in the SR1, 5.13% in the SR2, and 3.23% in the SR3, and bicyclogermacrene was found in a percentage of 3.20% in the SR1, 3.17% in the SR2, and 5.81% in the SR3. Kouklina et al. analyzed the EOs of the *S. raeseri* from two different localities of Greece and identified *(Z)*-9-octadecen-1-ol, (12.4% in one sample) and bicyclogermacrene (11.20% and 4.60%, respectively) [13]. In addition, in our samples, spathulenol was identified in a percentage of 6.46% in the SR1, 0.81% in the SR2, and 5.03% in the SR3. Pljevljakušic et al. also uncovered, the presence of spanthulenol (5.00–15.00%), in the aerial parts of four samples (four different stages of flowering) of *S. raeseri*, which were cultivated in Serbia [16].

### 4.3. Sideritis syriaca

The EOs of the *S. syriaca* from three different localities of Crete Island (Southern Greece—SS1, SS2, and SS3) presented variable chemical composition. However, 25 components were identified in all of the three samples: hexanol; benzaldehyde; 1-octen-3-ol; *(2E,4E)*-heptadienal; benzeneacetaldeyde; *cis*-linalool oxide; linalool; *cis*-*p*-menth-2-en-1-ol; *α*-campholenal; *trans*-pinocarveol; *p*-mentha-1,5-dien-8-ol; terpinen-4-ol; cryptone; *α*-terpineol; myrtenol; myrtenal; verbenone; *trans*-carveol; carvone; carvacrol; 4-methoxy-acetophenone; *(E)*-*β*-damascenone; *(E)*-caryophyllene; spathulenol; and caryophyllene oxide.

Aligiannis et al. reported the presence of 1-octen-3-ol in the essential oil of *S. syriaca* in a percentage of 2.27% [8]. In the essential oil of the SS1, 1-octen-3-ol was the main compound, identified in a percentage of 17.90%, and in the EOs of the SS2 and SS3, it was identified in a lower percentage (2.41% and 3.94%, respectively). Furthermore, Aligiannis et al. identified carvacrol in a percentage of 33.68% [8]. In the EO of the SS2, carvacrol was the major compound (19.75%), and in the EOs of the SS1 and SS3, it was identified in a percentage of 4.52% and 0.19%, respectively. In addition, in a previous study in the EO of the *S. syriaca* from Bulgaria, the presence of *α*-pinene, *β*-pinene, bicyclogermacrene, and D-germacrene (18.2%, 3.00%, 3.30%, 4.80%, respectively) was reported [17]. These components were also identified in the EOs of our samples: *α*-pinene was identified in the SS1 in a proportion of 0.40%, 0.53% in the SS2, and 1.62% in the SS3; *β*-pinene was identified in a proportion of 1.26% in the SS3; bicyclogermacrene was identified in a proportion of 2.66% in the SS3; and D-germacrene was identified in the EOs of the SS2—3.41%—and SS3—7.22%. According to Tirillini et al. [18], the major component of the *S. syriaca*, collected from Central Italy, was hexadecanoic acid (31.10%), which was also identified in the EO of the SS2 (1.03%). In addition, caryophyllene oxide (4.00%) was reported, which was also determined in the EOs of our samples: SS1 contained 0.37%, SS2 contained 4.21%, and SS3 contained 1.37%. Finally, Kouklina et al. reported in the EO of *S. syriaca* the presence of *β*-phellandrene (18.5%), kaur-15-ene (17.3%), hexadecanoic acid (5.9%), *α*-bisabolol (4.8%), and *α*-pinene (4.6%) [11]. In our samples, we identified *α*-pinene, (0.40% in the SS1, 0.53% in the SS2, and 1.62% in the SS3), *β*-phellandrene (SS2: 6.64% in the SS2 and 4.29% in the SS3), and hexadecanoic acid (1.03% in the SS2).

## 5. Conclusions

The EOs of three *Sideritis* species cultivated in different areas in Greece, namely, *S. scardica* (SSC1, SSC2, and SSC3), *S. raeseri* (SR1, SR2, and SR3), and *S. syriaca* (SS1, SS2, and SS3), were analyzed by GC-MS analysis and showed considerably different variability in their chemical composition. Comparing their contents, 1-octen-3-ol, linalool, trans-pinocarveol, p-mentha-1,5-dien-8-ol, α-terpineol, myrtenol, and verbenone were revealed as the common constituents in the nine EOs; however, they were estimated in different percentages among the samples.

Furthermore, it was found that the EOs of the *S. scardica* and *S. raeseri* from three different regions of Greece and the *S. syriaca* from three different localities of Crete Island, in Southern Greece, yielded different major components.

The essential oil’s chemical variability in relation to the examined plant species was presented using a dendrogram dividing the samples into two main groups and of one group to six subgroups. Among the same species of different geographical origins, the observed qualitative and quantitative differences can be attributed to abiotic factors such as soil, temperature, humidity, rainfall, and altitude, which influence the biosynthetic pathways of certain essential oil constituents.

The present work presents new data for the EOs’ chemical composition of the cultivated *S. scardica*, *S. raeseri*, and *S. syriaca* of different geographical origins in Greece, which therefore contribute to the existing literature.

## Figures and Tables

**Table 1:** Geographical origin of *Sideritis* samples.

Sample	Species	Geographical Origin	Latitude	Longitude	Elevation (m)	Voucher Number
SSC1	*Scardica*	Mount Olympus, Central Greece	39°59′	22°23′	900	012276
SSC2	*Scardica*	Mount Mainalo, Peloponnesos, Southern Greece	37°57′	22°25′	900	012279
SSC3	*Scardica*	Kastoria, Northern Greece	40°54′	21°34′	800	012293
SR1	*Raeseri*	Mount Othrys, Central Greece	39°06′	22°21′	780	012277
SR2	*Raeseri*	Kastoria, Northern Greece	40°54′	21°34′	800	012294
SR3	*Raeseri*	Elassona, Larissa, Central Greece	40°02′	22°05′	625	012295
SS1	*Syriaca*	White Mountains (Lefka Ori), Crete island, Southern Greece	35°23′	24°08′	700	012278
SS2	*Syriaca*	Anopoli Sfakion, Crete island, Southern Greece	35°22′	24°08′	650	012291
SS3	*Syriaca*	Omalos Chanion, Crete island, Southern Greece	35°232′	23°91′	1100	012292

**Table 2:** Essential oil yields % (*v*/*w*) of *Sideritis* samples.

Sample	Oil Yield % (*v*/*w*)
SSC1	0.02
SSC2	0.03
SSC3	0.05
SR1	0.02
SR2	0.05
SR3	0.10
SS1	0.02
SS2	0.05
SS3	0.01

**Table 3:** Chemical composition (%) of the essential oils of *Sideritis* samples.

No	Compound	RI Exp ^a^	RI Lit ^a^	SSC1 ^b^	SSC2 ^b^	SSC3 ^b^	SR1 ^b^	SR2 ^b^	SR3 ^b^	SS1 ^b^	SS2 ^b^	SS3 ^b^
1	*(E)*-2-Hexenal	846	846	- ^c^	8.75	-	-	-	-	-	-	-
2	Hexanol	860	863	-	3.59	1.89	-	1.50	0.15	16.23	0.87	9.84
3	Heptanal	897	901	-	-	-	-	-	-	2.89	0.82	1.47
4	*2,4-(E,E)*-Hexadienal	903	907	0.57	0.30	-	-	0.22	-	-	-	0.80
5	*α*-Thujene	926	924	-	-	-	-	0.20	0.75	0.89	-	-
6	*α*-Pinene	933	932	-	1.12	3.86	0.14	5.33	4.46	0.40	0.53	1.62
7	Benzaldehyde	952	952	1.96	0.97	2.04	-	1.44	0.32	5.83	2.07	6.65
8	Hexanoic acid	967	967	-	0.06	-	-	-	-	0.70	-	-
9	1-Octen-3-ol	974	974	7.91	37.28	13.99	1.32	6.14	1.83	17.90	2.41	3.94
10	*β*-Pinene	976	974	-	-	1.43	-	7.50	3.00	-	-	1.26
11	5-Hepten-2-one, 6-methyl-	980	981	-	-	-	-	-	-	-	-	0.38
12	Myrcene	988	988	-	-	-	-	0.52	1.45	-	-	-
13	3-Octanol	991	988	-	-	0.37	-	-	-	-	-	-
14	*α*-Phellandrene	1004	1002	-	-	-	-	-	2.90	-	-	-
15	*(2E,4E)*-Heptadienal	1007	1005	-	1.07	0.21	-	0.38	-	2.80	1.03	1.57
16	3-*δ*-Carene	1010	1008	-	-	-	-	-	3.73	-	0.38	0.30
17	*α*-Terpinene	1015	1014	-	-	-	-	0.09	1.06	-	0.17	-
18	*p*-Cymene	1022	1020	-	-	-	0.14	0.09	2.79	-	0.32	0.12
19	Benzyl alcohol	1026	1026	-	-	0.76	-	-	-	1.39	-	-
20	D-Limonene	1027	1024	-	-	-	-	3.32	4.44	-	-	-
21	*β*-Phellandrene	1028	1025	-	-	-	-	-	-	-	6.64	4.29
22	Eucalyptol	1029	1026	-	-	-	-	-	-	0.84	-	-
23	*β*-Ocimene	1034	1032	-	-	-	-	-	1.92	-	0.10	-
24	Benzene acetaldehyde	1035	1036	16.42	2.05	1.93	0.13	1.24	-	3.58	3.16	5.15
25	*(E)*-2-Octen-1-al	1053	1049	-	0.10	-	-	-	-	-	0.18	-
26	*γ*-Terpinene	1056	1054	-	-	-	-	0.29	1.05	-	0.37	-
27	Acetophenone	1061	1059	0.15	-	-	-	-	-	0.48	-	0.32
28	2-Methyl-,benzaldehyde	1061	-	-	-	-	-	-	-	0.34	0.56	-
29	Octanol	1063	1063	-	-	-	-	0.29	-	1.36	-	1.29
30	*cis*-Linalool oxide	1067	1067	-	-	-	0.14	0.24	-	0.37	0.29	0.28
31	Isopinocampheol	1071	-	-	-	-	0.08	-	-	-	-	-
32	Tetramethyl-pyrazine	1081	1081	-	-	-	-	-	-	0.30	-	-
33	*trans*-Linalool oxide	1084	1084	0.21	-	-	-	-	-	-	-	-
34	Terpinolene	1086	1086	-	-	-	-	0.59	0.57	-	-	-
35	Linalool	1096	1095	1.33	4.32	1.47	1.14	1.65	0.53	4.04	1.14	2.70
36	Nonanal	1102	1100	-	0.03	-	0.21		0.19	-	-	1.00
37	Phenylethyl Alcohol	1110	1106	5.71	0.33	-	-	-	-	-	-	0.39
38	*endo*-Fenchol	1111	1114	-	-	-	-	-	-	0.12	-	-
39	*cis*-*p*-Menth-2-en-1-ol	1119	1118	-	-	-	-	-	-	0.41	1.00	0.40
40	*α*-Campholenal	1123	1122	-	0.31	-	0.24	0.11	0.14	0.17	0.13	0.13
41	Nopinone	1132	1135	0.53	0.38	-	0.10	1.92	0.15	0.48	-	0.27
42	*cis*-*p*-Mentha-1(7),8-dien-2-ol	1133	-	-	-	-	-	-	-	-	0.25	-
43	*trans*-Pinocarveol	1136	1135	0.66	2.07	1.02	0.82	3.60	0.56	1.66	1.01	1.11
44	*cis*-Verbenol	1142	1137	-	-	1.29	0.12	1.52	0.28	1.00	1.42	0.20
45	*trans*-Verbenol	1144	1140	-	1.36	-	-	1.15	-	0.97	-	0.73
46	Sabina ketone	1151	1154	-	-	-	-	0.56	-	-	-	-
47	Pinocarvone	1158	1160	-	0.85	-	0.47	1.15	0.22	0.58	-	0.46
48	*p*-Mentha-1,5-dien-8-ol	1167	1166	2.64	4.55	1.82	0.74	2.84	0.41	1.36	1.22	0.85
49	Terpinen-4-ol	1174	1174	-	0.44	0.84	0.31	2.51	0.92	2.59	4.44	1.56
50	*p*-Methyl-acetophenone	1177	-	-	-	-	-	-	-	0.28	-	-
51	*p*-Cymen-8-ol	1183	1179	0.56	0.27	-	0.13	-	-	-	-	-
52	Cryptone	1184	1183	-	-	-	-	-	0.24	2.67	6.21	2.55
53	*α*-Terpineol	1187	1186	1.41	1.96	1.76	1.10	2.23	1.14	4.92	5.73	2.78
54	Methyl salicylate	1188	1190	0.37	0.55	-	-	-	-	-	-	-
55	Myrtenol	1194	1194	0.48	0.19	0.52	0.52	1.37	0.29	0.59	0.15	0.41
56	Myrtenal	1195	1195	-	-	0.39	-	2.00	0.29	0.89	0.28	0.48
57	Verbenone	1204	1204	1.55	1.12	0.42	0.16	1.08	0.20	0.58	0.60	0.24
58	Eucarvone	1206	-	-	-	-	0.07	-	-	-	-	-
59	*trans*-Carveol	1217	1215	0.37	-	-	0.09	0.66	0.16	0.42	0.45	0.23
60	Nerol	1224	1227	-	-	-	-	-	-	0.22	-	-
61	*cis*-Carveol	1226	1226	-	-	-	-	0.08	-	-	-	-
62	*cis*-3-Hexenyl-*α*-methylbu-tyrate	1228	1229	-	-	-	0.04	-	-	-	-	-
63	Pulegone	1233	1233	-	1.96	0.47	-	0.24	-	0.53	-	-
64	Cumin aldehyde	1233	-	-	-	-	-	-	0.14	-	0.65	0.38
65	Carvone	1240	1239	-	0.19	-	0.16	0.41	0.18	0.47	0.29	0.20
66	Geraniol	1251	1249	1.07	-	-	0.12	0.35	-	0.46	-	-
67	*trans*-2-Decenal	1258	-	-	-	-	0.02	-	-	-	-	-
68	Geranial	1264	1264	-	-	-	0.01	-	-	-	-	-
69	Nonanoic acid	1275	1267	3.87	-	-	0.66	0.22	-	0.87	-	-
70	Bornyl acetate	1281	1254	-	-	-	0.18	0.18	-	-	-	-
71	Thymol	1286	1289	0.56	-	1.16	0.18	0.29	-	0.11	-	-
72	Carvacrol	1296	1298	7.98	-	19.91	1.54	3.88	0.22	4.52	19.75	0.19
73	2-Methoxy-4-vinylphenol	1303	-	2.74	-	-	-	-	-	-	-	-
74	2-Methylpropyl ester-Benzoic acid	1324	-	-	-	-	0.03	-	-	-	-	
75	*δ*-Elemene	1338	1335	-	-	-	0.40	-	-	-	-	0.16
76	4-Methoxy-acetophenone	1343		-	-	-	-	-	-	1.86	0.26	0.86
77	*α*-Cubebene	1344	1345	-	-	-	0.10	-	-	-	-	-
78	Eugenol	1351	1356	21.52	3.83	1.70	0.31	0.36	-	1.30	-	0.35
79	*γ*-Nonanolactone	1354	-	0.27	-	-	-	-	-	-	-	-
80	Ylangene	1373	1373	-	-	-	0.60	-	-	-	-	-
81	*α*-Copaene	1378	1374	-	-	0.41	-	0.18	0.28	-	0.12	1.42
82	*(E)*-*β*-Damascenone	1380	1383	-	-	-	0.20	-	-	0.28	0.22	0.26
83	*β*-Bourbonene	1386	1387	-	-	-	0.12	0.11	-	-	-	-
84	4-(2,2-Dimethyl-6-methyle-ne-cyclohexyl)-2-butanone,	1390	-	-	-	-	0.13	-	-	-	-	-
85	*β*-Elemene	1392	1389	-	-	-	-	0.22	-	-	-	0.43
86	4-Dimethyl-*γ*-benzenebuta-nal	1396	-	-	-	-	0.24	-	-	-	-	-
87	*α*-Gurjunene	1404	1409	-	-	-	0.06	-	-	-	-	0.56
88	4-(2,6,6-Trimethyl-1,3-cycl-ohexadien-1-yl)-2-butanone	1409	-	-	-	-	0.30	-	-	-	-	-
89	*α*-Cedrene	1415	1410	-	-	-	-	-	-	-	-	0.14
90	*(E)*-Caryophyllene	1421	1417	-	-	3.40	4.03	4.13	0.91	0.33	9.09	10.18
91	*β*-Copaene	1426	1430	-	-	-	0.07	-	-	-	-	-
92	*α*-Bergamotene	1430	1432	-	-	-	0.03	-	-	-	-	-
93	3-Methyl,-1-Butanol, benzoate	1433	-	-	-	-	0.20	-	-	-	-	-
94	*(Z)*-β-Famesene	1437	1440	-	-	-	0.04	-	-	-	-	-
95	*cis*-Muurola-3,5-diene	1445	1448	-	-	-	0.34	-	0.16	-	-	-
96	*(E)*-β-Farnesene	1454	1454	-	-	1.34	0.56	1.29	0.43	-	1.20	1.26
97	Alloaromadendrene	1455	1458	-	-	-	0.09	-	-	-	-	-
98	2,6,10-Trimethyltridecane	1461	-	-	-	-	0.08	-	-	-	-	-
99	*epi*-*β*-Caryophyllene	1463	1464	-	-	-	-	-	0.13	-	-	-
100	*α*-Acoradiene	1466	1464	-	-	-	-	-	-	-	-	0.60
101	*trans*-Cadina-1(6),4-diene	1468	-	-	-	-	0.15	-	-	-	-	-
102	Pentyl benzoate	1471	1476	-	-	-	0.12	-	-	-	-	-
103	*γ*-Curcumene	1479	1479	-	-	-	-	-	-	-	-	1.62
104	Phenyl ethyl 2-methylbutanoate	1480	1486	-	-	-	0.06	-	-	-	-	-
105	D-Germacrene	1482	1484	-	0.87	3.80	1.10	5.25	0.39	-	3.41	7.22
106	*γ*-Amorphene	1487	1495	-	-	-	0.47	-	-	-	-	-
107	*α*-Zingiberene	1494	1493	-	-	-	-	-	-	-	-	2.51
108	*α*-Muurolene	1494	1500	-	-	-	0.01	-	-	-	-	-
109	Bicyclogermacrene	1497	1500	-	-	3.33	3.20	3.17	5.81	-	-	2.66
110	*β*-Bisabolene	1505	1505	-	-	-	0.74	0.53	0.98	-	-	-
111	*β*-Curcumene	1511	1514	-	-	-	-	-	-	-	-	1.20
112	Cubebol	1515	1514	-	-	-	-	-	0.09	-	-	-
113	*trans*-Calamenene	1522	1521	-	-	-	0.65	-	-	-	-	-
114	*δ*-Cadinene	1523	1522	-	-	-	0.94	0.45	-	-	-	2.91
115	*trans*-Cadina-1,4-diene	1528	-	-	-	-	0.72	-	0.42	-	-	-
116	*α*-Calacorene	1536	1544	-	-	-	0.37	-	-	-	-	-
117	*β*-Calacorene	1556	-	-	-	-	0.08	-	-	-	-	-
118	*trans*-Nerolidol	1560	1561	-	-	-	0.09	-	0.14	-	-	-
119	*(Z)*-3-Hexen-1-ol, benzoate	1568	-	-	-	-	0.58	-	0.44	-	-	-
120	Spathulenol	1578	1577	-	-	1.02	6.46	0.81	5.03	0.33	1.12	0.91
121	Caryophyllene oxide	1583	1582	-	-	1.17	2.92	0.77	1.11	0.37	4.21	1.37
122	Globulol	1590	1590	-	-	-	0.46	-	-	-	-	-
123	Viridiflorol	1592	1592	-	-	-	0.97	-	-	-	-	-
124	Rosifoliol	1601	1600	-	-	-	0.11	-	-	-	-	-
125	Humulene epoxide II	1604	1608	-	-	-	0.52	-	-	-	0.50	-
126	Isospathulenol	1628	-	-	-	-	0.35	-	-	-	-	-
127	Caryophylladienol II	1634	-	-	-	-	0.21	-	-	-	0.48	-
128	tau.-Muurolol	1639	1640	-	-	1.20	2.02	0.09	-	-	-	-
129	Bisabolol oxide II	1650	-	-	-	-	0.51	-	0.41	-	-	-
130	*α*-Cadinol	1653	1652	-	-	-	-	0.10	-	-	-	-
131	*(Z,Z)*-1,8,11-Heptadecatrie-ne	1659	-	-	-	-	0.24	-	-	-	-	-
132	*(Z,Z,Z)*-,1,8,11,14-Heptade-catetraene	1663	-	-	-	-	0.28	-	-	-	-	-
133	Valeranone	1668	1674	-	-	-	1.82	-	0.65	-	-	-
134	Aromadendrene oxide-(1)	1671	-	-	-	-	0.47	-	-	-	-	-
135	*α*-Bisabolol	1683	1683	-	-	-	3.24	0.21	4.56	-	-	-
136	Pentadecanal	1712	-	-	-	-	0.16	-	-	-	-	-
137	*trans*-Nuciferol	1718	1713	-	-	-	0.11	-	-	-	-	-
138	Benzyl benzoate	1758	1759	1.31	-	-	0.45	0.84	0.26	0.16	0.46	-
139	Tetradecanoic acid	1761	-	-	-	-	0.10	-	-	-	-	-
140	6,10,14-Trimethyl-2-Pentadecanone	1838	-	-	-	-	0.55	0.19	-	-	-	-
141	Geranyl-*α*-terpinene ^d^	1845	-	-	-	-	0.58	-	-	-	-	-
142	Benzyl salicylate	1859	1864	-	-	-	0.05	-	-	-	-	-
143	Geranyl-*α*-terpinene ^d^	1863	-	-	-	-	0.23	-	-	-	-	-
144	*(E,E)*-7,11,15-Trimethyl-3-methylene-hexadeca-1,6,10,14-tetraene	1902	-	-	-	-	0.40	-	-	-	-	-
145	Geranyl-*α*-terpinene ^d^	1910	-	-	-	-	1.52	-	-	-	-	-
146	Geranyl-*α*-terpinene ^d^	1918	-	-	-	-	0.30	-	-	-	-	-
147	Cembrene	1922	1937	-	-	-	-	-	1.39	-	-	-
148	Isopimara-9(11),15-diene	1926	-	-	-	-	0.17	-	-	-	-	-
149	Pimaradiene	1936	-	-	-	-	0.85	-	-	-	-	-
150	Geranyl-p-cymene	1952	-	-	1.15	1.88	3.60	0.20	3.13	-	0.21	0.44
151	Sandaracopimaradiene	1955	1968	-	-	-	0.17	-	-	-	-	-
152	Geranyl-*α*-terpinene ^e^	1960	-	-	-	-	4.58	-	-	-	-	-
153	Hexadecanoic acid	1962	-	-	-	4.54	3.10	4.16	1.96	-	1.03	-
154	Geranyl-*α*-terpinene ^e^	1968	-	-	-	-	-	-	5.84	-	-	-
155	*(Z,Z)*-Geranyl linalool	1977	-	-	-	-	1.75	-	-	-	-	-
156	Kaur-15-ene	1987	1997	-	-	-	1.13	0.20	0.20	-	-	-
157	*(E,Z)*-Geranyl linalool	1990	1987	-	-	-	0.77	-	-	-	-	-
158	13-*epi*-Manoyl oxide	2004	-	-	-	-	1.20	-	-	-	-	-
159	*(E,E)*-Geranyl linalool	2018	2026	-	-	-	0.14	-	-	-		-
160	*(Z)*-9-Octadecen-1-ol	2059	-	-	-	-	12.00	5.13	3.23	-	-	-
161	*(E)*-9-Octadecen-1-ol	2069	-	-	3.04	-	-	-	-	-	-	-
162	1-Heneicosene	2088	2100	-	-	-	0.36	-	-	-	-	-
163	5-(7a-Isopropenyl-4,5-dimethyl-octahydroinden-4-yl)-3-methyl-pent-2-en-1-ol	2094	-	-	-	-	1.01	-	-	-	-	-
164	Linoleic acid	2131	2132	-	-	2.11	-	2.23	1.80	-	-	-
165	Cembrenol	2165	-	-	-	-	-	-	1.29	-	-	-
	Monoterpene hydrocarbons (%)			-	1.12	5.29	0.28	17.93	**28.12**	1.29	8.51	7.59
	Oxygenated monoterpenes (%)			36.5	26.86	**35.46**	9.33	**31.91**	6.26	**39.98**	**49.13**	24.91
	Sesquiterpene hydrocarbons (%)			-	0.87	12.28	15.47	15.33	9.51	0.33	13.82	**32.87**
	Oxygenated sesquiterpenes (%)			-	3.04	3.39	**35.1**	8.14	15.92	0.86	6.77	2.28
	Diterpenes (%)			-	1.15	1.88	18.76	0.40	11.85	-	0.21	0.44
	Others (%)			**45.65**	**52.02**	25.15	5.21	16.29	6.06	49.38	8.49	25.51
	Total intedified (%)			**82.15**	**85.06**	**83.45**	**84.15**	**90**	**77.72**	**91.84**	**86.93**	**93.6**

^a^ RIexp: experimental retention index calculated against C7-C24 *n*-alkanes on the OPTIMA-5MS column; RIlit: literature retention index [12]. ^b^ SSC1: *S. scardica* from Olympus; SSC2: *S. scardica* from Mainalo; SSC3: *S. scardica* from Kastoria; SR1: *S. raeseri* from Othrys; SR2: *S. raeseri* from Kastoria; SR3: *S. raeseri* from Elassona; SS1: *S. syriaca* from Lefka Ori; SS2: *S. syriaca* from Anopoli Sfakion; SS3: *S. syriaca* from Omalos. ^c^ not identified. ^d^ correct isomer did not identify. ^e^ correct isomer did not identify.

## Data Availability

The data presented in this study are available upon request from the corresponding author.
